# Exploration of the Molecular Basis of Forsythia Fruit in the Prevention and Treatment of Cholestatic Liver Injury through Network Pharmacology and Molecular Docking

**DOI:** 10.3390/nu15092065

**Published:** 2023-04-25

**Authors:** Ke Fu, Yanzhi Li, Shu Dai, Yunxia Li

**Affiliations:** State Key Laboratory of Southwestern Chinese Medicine Resources, Key Laboratory of Standardization for Chinese Herbal Medicine, Ministry of Education, School of Pharmacy, Chengdu University of Traditional Chinese Medicine, Chengdu 611137, China; fuke1993@stu.cdutcm.edu.cn (K.F.); liyanzhi@stu.cdutcm.edu.cn (Y.L.);

**Keywords:** cholestasis, forsythia fruit, forsythoside A, network pharmacology, molecular docking, TLR4/NF-κB pathway

## Abstract

Forsythia fruit, edible fruit of *Forsythia suspensa* (Thunb.) Vahl, which has been found to be effective in treating cholestasis. However, its key component for alleviating cholestasis has not been determined. In this study, four representative active ingredients in forsythia fruit were selected. Through network pharmacology and molecular docking technology, we tried to find the key component for its treatment of cholestasis. Furthermore, the model of cholestasis in mice was established to verify the protective effect of the key component on cholestasis. Network pharmacology and molecular docking showed that forsythoside A (FTA) is the key component of forsythia fruit in the treatment of cholestasis. In vivo experiments revealed that FTA treatment could alleviate liver injury, dysfunction, and collagen deposition induced by cholestasis in mice. At the same time, FTA treatment inhibited inflammatory factor release and fibrosis-related factor expression. In addition, FTA treatment also reduced MMP-2, TLR4, MYD88, NF-κB p65, and *p*-NF-κB p65 protein expression. In conclusion, FTA, a key component of forsythia fruit, alleviated liver damage and fibrosis caused by cholestasis via inhibiting the TLR4/NF-κB pathway, extracellular matrix accumulation, and inflammatory cytokine expression. The research results could provide a scientific reference for the development of forsythia fruit as a drug or functional food to prevent and treat cholestasis.

## 1. Introduction

Cholestasis or cholestatic liver disease (CLD) is the accumulation of excess bile acid and abnormal metabolites in the body induced by bile duct injury, leading to apoptosis and necrosis of bile duct cells and hepatocytes. If allowed to develop without intervention, it may eventually develop into liver fibrosis and cirrhosis, or even liver failure [1,2]. Chronic CLD is typified by primary biliary cholangitis (PBC) and primary sclerosing cholangitis (PSC), with pruritus as the most common complication [3,4]. Unfortunately, the pathophysiological processes of these diseases have not yet been scientifically resolved. Evidence suggests that a genetic cause of CLD has been identified as the underlying cause of liver disease in “idiopathic” children and adults [5]. Currently, although ursodeoxycholic acid (UDCA) has been used as a clinical treatment for most CLD patients, a portion of PBC patients (approximately one-third of the total) have no obvious response to UDCA treatment. Obechoic acid (OCA) can be used in non-responders of UDCA, but more clinical evidence is needed to reveal the safety and efficacy of this new therapeutic drug [6,7]. In addition, there is no clinical evidence of any drugs that can change the course of PSC. Therefore, PSC patients will be at risk of disease progression [8]. In view of this, more effective drugs should be mined to deal with CLD, especially for PSC.

Herbal remedies are the first drugs used by humans due to the fact that plants can produce a variety of secondary metabolites that have medicinal effects on humans (e.g., antiviral, antioxidant, anticancer, etc.) [9,10,11]. According to the World Health Organization, globally, more than 75% of the population still relies on herbal medicine resources [12]. At present, various drugs used to prevent and treat major diseases in clinical practice should be attributed to medicinal plants. To take a few classic examples, artemisinin can be used clinically to treat malaria by damaging the structure of the plasmodium nuclear membrane and mitochondrial outer membrane [13]. Paclitaxel, derived from the medicinal plant Yew, achieves its anticancer effects by inhibiting mitosis and triggering apoptosis in cancer cells [9]. Today, scholars have been committed to exploring the active components of plants that exert therapeutic effects to further determine the molecular basis of medicinal plants for treating diseases. Forsythia fruit, the fruit of *Forsythia suspensa* (Thunb.) Vahl (FS), is an aromatic and edible fruit. In the 2020 edition of the Ch.P, the fruits were identified as the medicinal part of FS. Extensive evidence shows that FS can effectively exert hepatoprotective effects through its anti-inflammatory, antioxidant, anti-fibrosis, and bile-promoting properties [14,15,16]. FS can alleviate intrahepatic cholestasis in young rats by inhibiting bile duct proliferation and inflammatory cell infiltration and improving liver function [17]. Clinical evidence shows that FS can effectively treat mild intrahepatic cholestasis of pregnancy by reducing the level of total bile acids and improving liver function and pruritus [18]. However, at present, the molecular basis of FS in alleviating cholestasis has not been revealed.

Due to the inclusion of multiple bioactive compounds in medicinal and edible plants, which can exert pharmacological effects through multiple pathways and targets, it is extremely difficult to elucidate their molecular basis and mechanism of action using traditional pharmacological methods. With the development of bioinformatics and systems biology, network pharmacology has been proposed as a new method for drug discovery [19]. Network pharmacology updates the traditional “one compound, one target” model of drug research to a “multiple components, network targets” model, which describes the interactions of multiple compounds with complex biological systems as networks [20,21], thereby providing an understanding of how biological systems, drugs, and diseases are interconnected [22]. The research paradigm and methodology of network pharmacology are well suited for the study of botanicals with complex active ingredients and action targets [23]. However, as network pharmacology is still in an immature stage of development, the existing databases still suffer from incomplete data and uncontrolled quality. Also, the results of different database algorithms vary considerably, so the predicted results of network pharmacology may not be fully convincing, thus requiring the application of other methods for validation [24,25]. Molecular docking techniques for drug design based on structure and in vivo or in vitro experiments in the real world are commonly used to validate the results of network pharmacology [26,27].

In this study, we first analyzed the possible key component of FS in the prevention and treatment of cholestasis via network pharmacology and molecular docking techniques. Then, a cholestasis mouse model was established to validate the effectiveness and molecular mechanisms of the key component to alleviate cholestasis. The aim of this study is to clarify the molecular basis of FS for the prevention and treatment of cholestasis and to lay a scientific foundation for the further development of functional foods and drugs for the treatment of cholestasis.

## 2. Materials and Methods

### 2.1. Network Pharmacology Research

#### 2.1.1. Prediction of Action Targets of FS

Four representative active ingredients (RAIs) (Phillyrin, FTA, Phillygenin, and Pinoresinol 4-O-beta-D-glucopyranoside) used for quality control under “FS” and “FS extract” in the Ch.P (2020) were selected for follow-up study. SMILES for RAIs were downloaded from the PubChem database (https://pubchem.ncbi.nlm.nih.gov/ (accessed on 2 June 2022)). Then they were entered into the SwissTargetPrediction (http://www.swisstargetprediction.ch/ (accessed on 2 June 2022)) and SuperPred (https://prediction.charite.de/index.php (accessed on 2 June 2022)) online platforms [28,29], using “Homo sapiens” as the study species for target prediction. Probability > 0 and probability ≥ 50% were used as the threshold filter of the SwissTargetPrediction and SuperPred platforms, respectively.

#### 2.1.2. Building a “Components-Targets” Network

Cholestasis targets were collected in the DisGeNET database (http://www.genecards.org/ (accessed on 13 July 2022)) and GeneCards database (http://www.genecards.org/ (accessed on 13 July 2022)) using the keyword “Cholestasis”, then integrating the data and de-duplicating them. Using the Venny_v2.1.0 platform (https://bioinfogp.cnb.csic.es/tools/venny/index.html (accessed on 13 July 2022)), Venn diagrams of the RAI targets and cholestasis targets were plotted, with the intersection being the target of the RAIs for cholestasis treatment. Subsequently, the relationships between the four RAIs and their targets for the treatment of cholestasis were collated and introduced into Cytoscape_v3.8.2 software to construct the “components-targets” network [30].

#### 2.1.3. Building a PPI Network 

The Gene Symbols of the targets of the RAIs for cholestasis treatment were imported into the STRING_v11.0 data platform (https://string-db.org/ (accessed on 13 July 2022)), and the PPI relationships between the targets were obtained using “Homo sapiens” as the study species. The PPI network was established via Cytoscape_v3.8.2 software [30], and network topology analysis was performed via the Network Analyzer plug-in. 

#### 2.1.4. GO and KEGG Analysis 

The Gene Symbols of the targets of the RAIs for cholestasis treatment were imported into the DAVID_v6.8 database (https://david.ncifcrf.gov/ (accessed on 13 July 2022)) [31,32], and the species “Homo sapiens” was selected for GO and KEGG enrichment analysis. Depending on the *p*-value from smallest to largest, we selected the top 25 results to draw an advanced bubble chart on the OmicShare website (https://www.omicshare.com/ (accessed on 13 July 2022)).

#### 2.1.5. Screening the Key Component for the Treatment of Cholestasis 

The top 25 signal pathways were selected from the outcomes of the KEGG pathway enrichment analysis, and the relationship between pathways and targets, as well as between targets and components, were sorted out and imported into Cytoscape_v3.8.2 software to construct a “components-targets-paths” network [30]. Meanwhile, the NetworkAnalyzer plug-in was used for network topology analysis. Then, based on the outcomes of the PPI network, “components-targets-paths” network, KEGG pathway, and GO biological process enrichment analysis, RAI key targets for the treatment of cholestasis were selected. Next, the key targets were used as the receptor and the RAIs were used as the ligand to conduct molecular docking studies, and the key component of FS treatment for cholestasis was screened out by referring to the results of molecular docking and related studies.

#### 2.1.6. Network Pharmacology Research of Key Component

Network pharmacological studies (as above) were conducted on the key component screened out above, including the construction of the PPI network, enrichment analysis of the KEGG pathway and GO biological process, and the main targets and pathways of key components for the treatment of cholestasis were screened out.

### 2.2. Molecular Docking Research

#### 2.2.1. Preparation of Ligands and Receptors

The 3D structure files of the RAIs and FTA downloaded from the PubChem database were used as ligands. At the same time, the structure files of targets were downloaded from the RCSB database (https://www.rcsb.org/ (accessed on 28 September 2022)) and were used as receptors, which were prepared using OpenBabel_v2.4.1, PyMol_v2.4 and Autodock_v1.5.6 software and converted into qdbqt format [33].

#### 2.2.2. The Operation of Molecular Docking

The center coordinates of the docking pocket of the receptor proteins with the original ligand were set with reference to the original ligand, and the receptor proteins without the original ligand were set with reference to the center coordinates of the active amino acid residues included in the UniProt database (https://www.uniprot.org/ (accessed on 28 September 2022)), with an exhaustiveness of 20, using Autodock Vina_v1.1.2 software for molecular docking [34].

### 2.3. Experimental Study In Vivo

#### 2.3.1. Reagents and Chemicals

The reagents and chemicals used in this study are shown in Table 1.

#### 2.3.2. Animals and Treatments

**Figure 1:**
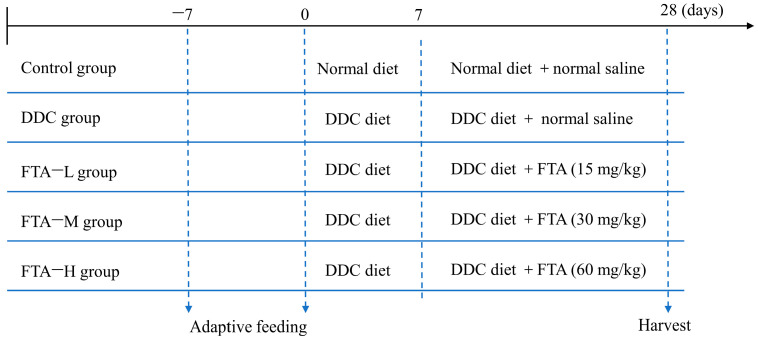
Experimental design.

The experimental subjects of this study were 30 8-week-old, male C57BL/6J mice (20 ± 2 g) purchased from Chengdu Dashuo Experimental Animal Co., Ltd. (Chengdu, China). Mice were housed in an SPF environment, and irradiated and sterilized feed and drinking water were freely available. A week later, the mice were fed adaptively and were randomly divided into five groups (*n* = 6): control group, DDC group, FTA low-dose (FTA-L), FTA medium-dose (FTA-M), and FTA high-dose (FTA-H) groups. The experimental protocol has been approved by the Chengdu University of Traditional Chinese Medicine (Grant No. SYXK-2020-124, Chengdu, China). Subsequently, in the DDC, FTA-L, FTA-M, and FTA-H groups, mice were fed a diet containing 0.1% DDC for four weeks, while the control group received a normal diet. After being fed the diet for one week, the mice in the FTA-L, FTA-M, and FTA-H groups were administrated with FTA (dissolved in physiological saline, 15, 30, and 60 mg/kg, 10 mL/kg), respectively, for three consecutive weeks. In the control group and the DDC group, mice received normal saline (10 mL/kg) for three weeks. After the experiment, the mice were euthanized by carbon dioxide, and blood and liver tissue were collected. The detailed experimental process is shown in Figure 1.

#### 2.3.3. Histological, Biochemical Assessments, and ELISA Analysis

A small piece of liver was fixed in a 10 mL EP tube containing 4% paraformaldehyde for 24 h. Subsequently, the fixed liver was then pruned, dehydrated, embedded, and sectioned for further staining. Pathological changes in the liver were evaluated using hematoxylin-eosin (HE) staining. Collagen deposition in liver was evaluated via Masson staining. In addition, serum AST and ALT were tested by assay kit to assess the change in liver function. At the same time, serum TNF-α, IL-6, and IL-1β were tested by ELISA kit to estimate systemic inflammation.

#### 2.3.4. Immunohistochemistry Analysis

According to the previous experimental methods of our research group [35], we used immunohistochemical staining to analyze α-SMA, F4/80, and *p*-NF-κB expression. Briefly, paraffin sections were made using mice liver tissue. The sections were placed in PBS (PH 7.4) to perform antigen repair. The sections were placed in a 3% hydrogen peroxide solution to block endogenous peroxidase. The sections were sealed at room temperature for 30 min using 3% BSA. Subsequently, the sections were incubated with primary and secondary antibodies and colored with DAB, respectively. Finally, dyed pictures were observed and collected under a microscope.

#### 2.3.5. Quantitative Real-Time PCR (qPCR) Analysis 

MMP-2, TNF-α, IL-1β, IL-6, and F4/80 mRNA expression levels were detected via qPCR. Briefly, a total RNA isolation kit was used to extract total RNA from liver tissues. Subsequently, the total RNA was then synthesized into stable cDNA using ChamQ SYBR qPCR Master Mix. Next, RT-qPCR was performed by adding ChamQ SYBR qPCR Master Mix according to the instructions of the reagent manufacturer. Table 2 shows all primers used in this study. Finally, we calculated the relative mRNA expression by 2^−ΔΔCT^.

#### 2.3.6. Western Blot

According to the previous experimental methods of our research group [36], MMP-2, α-SMA, TLR4, MYD88, NF-κB p65, and *p*- NF-κB p65 were tested via Western blot. Briefly, total protein was extracted from liver tissues and inactivated at 100 °C. Subsequently, electrophoresis, membrane transfer, and other operations were carried out to transfer the protein to the stable PVDF membrane. Then, the membrane was incubated in diluted MMP-2, α-SMA, TLR4, MYD88, NF-κB p65, and *p*-NF-κB p65 primary antibody (1:1000) and diluted secondary antibody (1:5000). Finally, the developer was dripped onto the membrane and the chemiluminescence was collected.

#### 2.3.7. Statistical Analysis

The data were compared among multiple groups by one-way ANOVA using SPSS 26.0 statistical software, and all results were expressed by mean ± SD. Histograms and heat maps were drawn using GraphPad Prism 9.0 software. Results were considered statistically significant when *p* < 0.05.

## 3. Results

### 3.1. The Targets of RAIs of FS in the Treatment of Cholestasis

**Figure 2:**
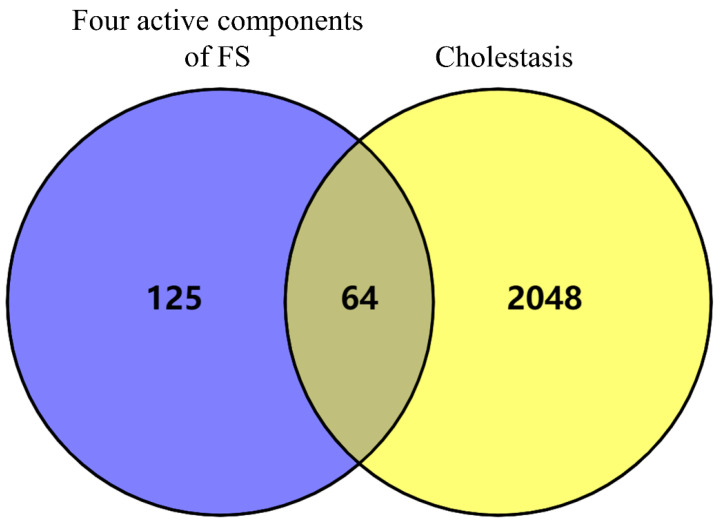
Venn diagram of the action targets of four active components of FS and the targets of cholestasis disease.

**Figure 3:**
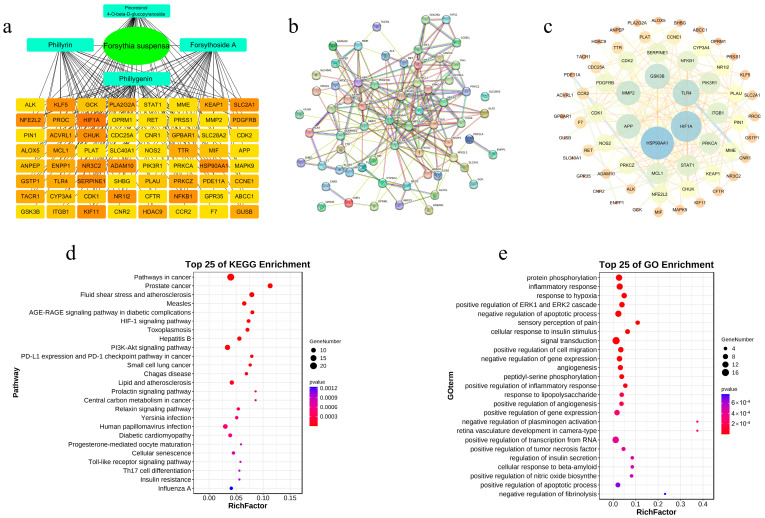
Network pharmacology research results of FS in the treatment of cholestasis. (**a**) “components-targets” network; (**b**) PPI network of four active components of FS in the treatment of cholestasis; (**c**) Network topology analysis of PPI network; (**d**) Top 25 results of KEGG pathway enrichment analysis; (**e**) Top 25 results of GO biological process enrichment analysis.

Basic information about the RAIs can be found in Table 3. A total of 189 targets were predicted for RAIs, and 2112 targets were predicted for cholestasis-related targets. The Venn diagram of the two was plotted on the OmicShare platform (Figure 2), and the results revealed that a total of 64 targets showed a critical role in cholestasis. In addition, 138 kinds of associations existed between the RAIs and 64 common targets, and the “components-targets” network was drawn (Figure 3a).

### 3.2. The Results of the PPI Network 

A total of 63 proteins were collected through the STRING_v11.0 database with 221 complex interactions between them (Figure 3b). Cyto-scape_v3.8.2 software was used to construct a PPI network of targets of the RAIs for cholestasis, as shown in Figure 3c. The median value of the PPI network degree was 5, and the median of median centrality was 0.0047219. Thirteen key targets were selected, namely HSP90AA1, HIF1A, TLR4, GSK3B, MMP2, APP, SERPINE1, NFKB1, PIK3R1, ITGB1, PRKCA, STAT1, and MCL1.

### 3.3. The Results of GO and KEGG Analysis 

Based on the KEGG enrichment analysis, 81 signaling pathways (*p* ≤ 0.05) were obtained, involving the pathway in cancer, HIF-1, PI3K-Akt, Prolactin, Relaxin, and Toll-like receptor signaling pathway, etc. Subsequently, the top 25 pathways were visualized and analyzed with advanced bubble plots (Figure 3d). In addition, GO bioprocess enrichment analysis yielded 131 entries (*p* ≤ 0.05), involving protein phosphorylation, inflammatory response, response to hypoxia, negative regulation of the apoptotic process, signal transduction, etc. The top 25 entries were visualized and analyzed with advanced bubble plots (Figure 3e).

### 3.4. Results of Screening Key Component

**Figure 4:**
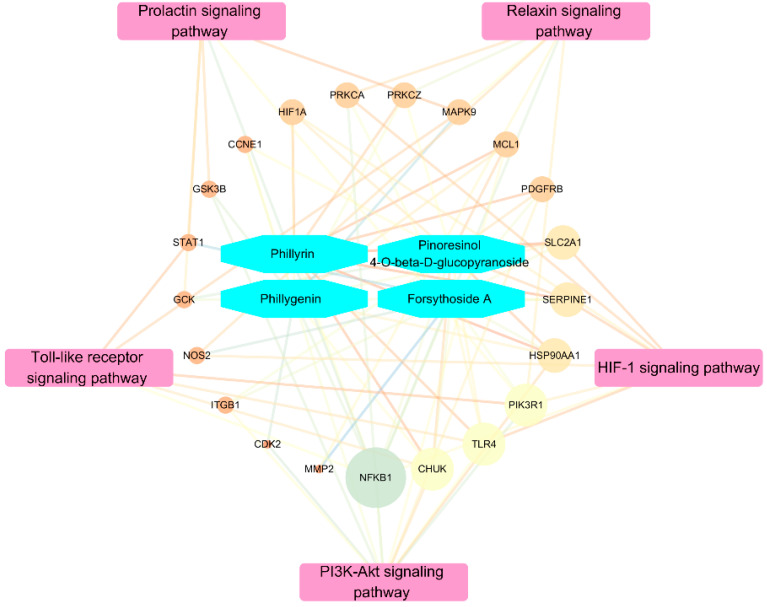
“Components-targets-pathways” network.

**Figure 5:**
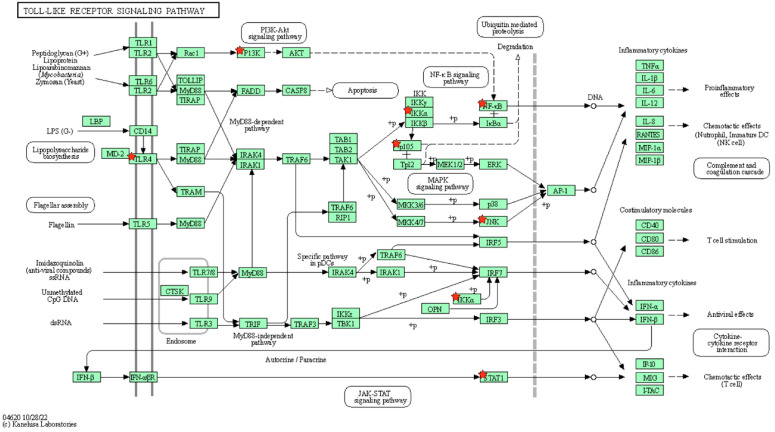
Effect of FS on toll-like receiver signaling pathway.

The results of the “components-targets-paths” network showed that TLR4’s degree value ranking was relatively high (Figure 4). At the same time, in the enrichment analysis of GO biological processes, the entries of TLR4 participating in the inflammatory response, positive regulation of inflammatory response, and positive regulation of gene expression were also relatively high. These data reflected that TLR4 may play a crucial role in the treatment of cholestasis by RAIs. TLR4 is mainly located in the Toll-like receptor signaling pathway. Based on the effect of the RAIs on this pathway (Figure 5), it can be inferred that the RAIs may exert their hepatoprotective effect mainly through the TLR4/NF-κB pathway. More importantly, NFKB1 ranked first in the “components-targets-paths” network. This result provided further evidence that the RAIs protects against liver injury via the TLR4/NF-κB pathway.

Therefore, TLR4 and NFKB1 were selected as receptors and the RAIs as ligands in this study. Molecular docking data revealed a relatively higher binding ability of FTA with TLR4 and NFKB1 (Table 4), suggesting that FTA was a key component in the treatment of cholestasis by RAIs. 

### 3.5. Network Pharmacology Study of Key Component FTA

**Figure 6:**
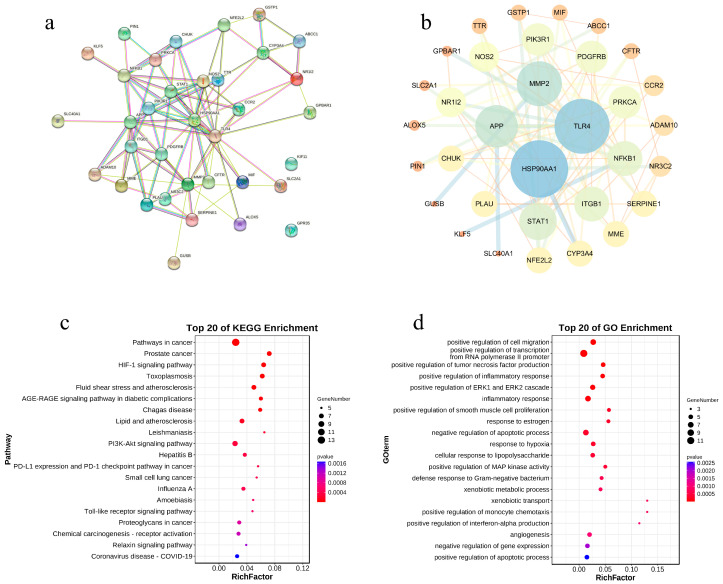
Network pharmacology research results of forsythoside A (FTA) in the treatment of cholestasis. (**a**) PPI network of targets for FTA treatment of cholestasis; (**b**) Network topology analysis of PPI network; (**c**) Top 20 results of KEGG pathway enrichment analysis; (**d**) Top 20 results of GO biological process enrichment analysis.

Our results showed that FTA had a total of 35 targets for cholestasis treatment, among which 33 proteins had interactions (Figure 6a). The constructed PPI network is shown in Figure 6b. Among them, HSP90AA1, TLR4, MMP2, APP, and NFKB1 were the crucial targets of FTA in the treatment of cholestasis. Based on KEGG enrichment analysis, 67 signaling pathways (*p* ≤ 0.05) were obtained, involving the HIF-1 signaling pathway, PI3K-Akt signaling pathway, Toll-like receptor signaling pathway, relaxin signaling pathway, etc. Subsequently, the top 20 pathways were visualized and analyzed with advanced bubble plots (Figure 6c). GO bioprocess enrichment analysis yielded 85 entries (*p* ≤ 0.05), involving positive regulation of cell migration, positive regulation of polymerase II promoter transcription, positive regulation of tumor necrosis factor production, positive regulation of inflammatory response, and positive regulation of ERK1 and ERK2 cascades. The top 20 entries were visualized and analyzed with advanced bubble plots (Figure 6d). It can be seen from these results that the network pharmacological results of FTA are similar to the network pharmacological results of an RAI, further supporting that FTA may be a key component in the treatment of cholestasis among the RAIs of FS.

### 3.6. Molecular Docking Study of FTA 

**Figure 7:**
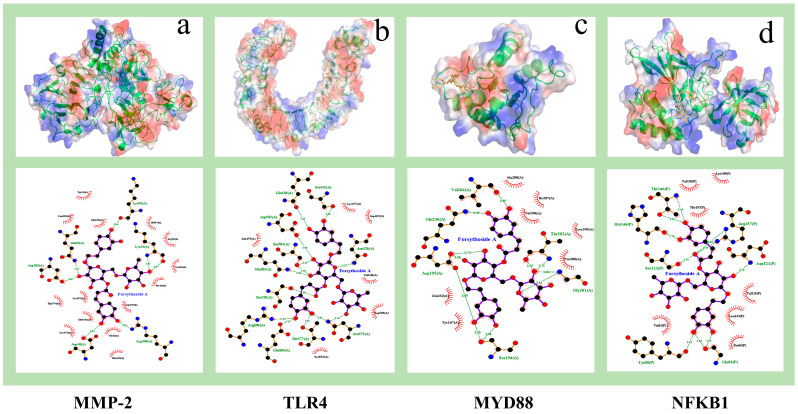
Conformation of ligand and receptor binding. The binding conformation and interaction force between FTA and MMP-2 (**a**), TLR4 (**b**), MYD88 (**c**), and NFKB1 (**d**).

On the one hand, we have proved that FS could ameliorate carbon tetrachloride-induced hepatic fibrosis in rats through the TRL4/NF-κB signaling [15]. On the other hand, when cholestasis occurs, it causes infiltration of inflammatory cells, thus causing the liver to suffer from inflammatory damage. Substantial evidence showed that inhibition of the TLR4/MYD88/NF-κB inflammatory pathway played a crucial role in the prevention and treatment of cholestasis [37,38,39]. In addition, MMP-2 has been proven to be highly expressed in cholestasis [40,41,42], suggesting that MMP-2 also played an important role in cholestasis. More importantly, by combining the outcomes of the PPI network, GO and KEGG analysis, we speculated that FTA could ameliorate cholestasis by acting on important targets such as TRL4, MYD88, NF-KB1, and MMP-2. Therefore, in this study, MMP2, TLR4, MYD88, and NFKB1 were selected for molecular docking with FTA. Table 5 shows the molecular docking results, and Figure 7 shows the conformation of ligands and receptors bounding. Referring to the relevant literature, it can be considered that the binding of ligands and receptors is better when the binding free energy is less than −5.0 kcal/mol [43]. Our outcomes revealed that the binding free energy of FTA and receptors was less than −5.0 kcal/mol, indicating that FTA may bind these receptors and regulate TLR4/Myd88/NF-κB signaling, thereby improving liver damage caused by cholestasis.

### 3.7. Validation of in Vivo Experiment

#### 3.7.1. FTA Improved DDC-Induced Liver Injury and Fibrosis

**Figure 8:**
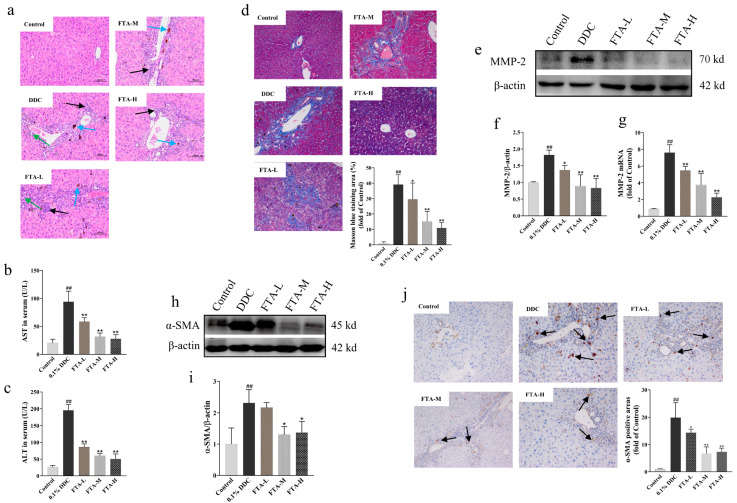
FTA alleviated DDC-induced liver injury and fibrosis. (**a**) HE staining. Black arrow: neutrophil infiltration; Blue arrow: pigment deposition; Green arrow: duct proliferation. (**b**,**c**) Serum AST and ALT levels measured by biochemical kits. (**d**) Masson staining. (**e**,**f**) Protein expression and quantitative analysis of MMP-2. (**g**) Liver MMP-2 mRNA expression was detected by RT-qPCR. (**h**,**i**) Protein expression and quantitative analysis of α-SMA. (**j**) α-SMA was detected by immunohistochemistry. ^##^ *p* < 0.01 vs. Control group, * *p* < 0.05 vs. 0.1% DDC group, ** *p* < 0.01 vs. 0.1% DDC group.

Four weeks after treatment with DDC, normal mice’s livers showed no pathological changes based on HE results. However, DDC induced the proliferation of connective tissue around the portal area of liver tissue, accompanied by a large amount of neutrophil infiltration, bile duct proliferation, pigment deposition, and hepatocyte necrosis (Figure 8a). In addition, DDC induced obvious abnormal changes in liver function, manifested by a significant increase in serum AST and ALT (Figure 8b,c). Fortunately, all the above abnormal changes caused by DDC were reversed by FTA. Next, in order to evaluate DDC-induced hepatic fibrosis in mice, a collagen deposition examination was performed using Masson staining. Our data showed that DDC caused obvious collagen deposition. However, hepatic collagen deposition of mice was effectively prevented by FTA intervention (Figure 8d). Subsequently, MMP-2 mRNA and protein levels were measured by RT-qPCR and WB, respectively. The results revealed that DDC induced a high expression of MMP-2 mRNA and protein (Figure 8e–g). This situation was significantly reversed through FTA intervention. Finally, Western blot and immunohistochemical staining were used to detect α-SMA expression. Similar to the above results, the high expression of α-SMA caused by DDC was reversed by FTA intervention (Figure 8h–j). In conclusion, our data revealed that FTA could effectively prevent DDC-induced liver injury and fibrosis.

#### 3.7.2. FTA Improved DDC-Induced Cholestatic Liver Injury by Blocking TLR4/MYD88/NF-κB Pathway

**Figure 9:**
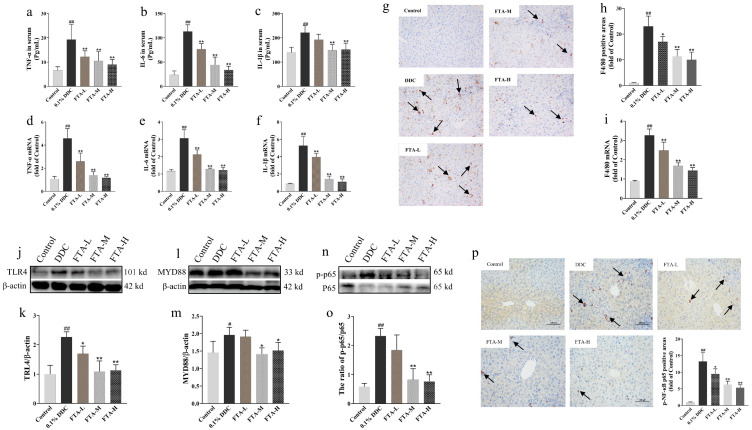
FTA alleviated DDC-induced cholestatic liver injury by inhibiting the release of inflammatory factors and the TLR4/MYD88/NF-κB signaling pathway. (**a**–**c**) Serum TNF-α, IL-6, and IL-1β levels measured by ELISA kits. (**d**–**f**) Liver TNF-α, IL-6, and IL-1β mRNAs expression detected by RT-qPCR. (**g**) F4/80 detected by immunohistochemistry. (**h**) Quantitative analysis of F4/80. (**i**) Liver F4/80 mRNA expression detected by RT-qPCR. (**j**–**o**) Proteins expression and quantitative analysis of TLR4, MYD88, and ratio of *p*-NF-κB p65/NF-κB p65. (**p**) *p*-NF-κB p65 detected by immunohistochemistry. ^#^ *p* < 0.05 vs. Control group, ^##^ *p* < 0.01 vs. Control group, * *p* < 0.05 vs. 0.1% DDC group, ** *p* < 0.01 vs. 0.1% DDC group.

To explore the inflammatory state of mice induced by DDC, firstly, the inflammatory state of the mouse system and liver was reflected by testing serum TNF-α, IL-6, and IL-1β levels and liver TNF-α, IL-6, and IL-1β mRNA expression. The data showed that DDC markedly increased serum TNF-α, IL-6, and IL-1β levels (Figure 9a–c). Meanwhile, TNF-α, IL-6, and IL-1β mRNA of the liver were highly expressed after DDC feeding (Figure 9d–f). More importantly, FTA intervention markedly decreased TNF-α, IL-6, and IL-1β expression in serum and liver. In addition, it has been reported that the F4/80 molecule was mainly expressed on macrophage surfaces and used as a marker of mature macrophages [44]. F4/80 was highly expressed in the body in the state of cholestasis [45]. In this research, F4/80 expression was determined by immunohistochemical staining and qPCR. Our data revealed that DDC could induce the high expression of F4/80. Conversely, FTA intervention could significantly inhibit the expression of F4/80 (Figure 9g–h). Next, in order to further verify the molecular mechanism of FTA preventing mice from inflammation. We tested TRL4, MYD88, NF-κB p65, and *p*-NF-κB p65 proteins expression. The data showed that DDC significantly increased TRL4, MYD88, NF-κB p65, and *p*-NF-κB p65 proteins (Figure 9j–o). This situation was reversed through FTA intervention. Finally, the *p*-NF-B p65 expression was detected by immunohistochemistry staining. Similar to the above outcomes, the high expression of *p*-NF-κB p65 caused by DDC was reversed by FTA intervention (Figure 9p). In conclusion, a cholestatic liver injury could be prevented by markedly blocking inflammatory factors expression and TRL4/MYD88/NF-B pathway activation with FTA intervention.

## 4. Discussion

In this study, by using a network pharmacology approach and a molecular docking technique, we explored the roles of FS and its key components in the prevention and treatment of cholestasis. First, the KEGG pathway enrichment analysis results suggested that FS may have strong impacts on the HIF-1, PI3K-Akt, relaxin, toll-like receptor, and other signaling pathways. Meanwhile, the network topology analysis results of the “component-target-pathway” network showed that NF-κB was closely related to these signaling pathways and the main active components of FS. In addition, we found that TLR4 played a central role in the PPI network and the “component-target-pathway” network. More importantly, the TLR4/NF-κB pathway is widely involved in the HIF-1, PI3K-Akt, and Toll-like receptor signaling pathways. These results suggested that the TLR4/NF-κB pathway may play a central role in the prevention and treatment of cholestasis in FS. On this basis, molecular docking outcomes further illustrated that the binding activity of the key components in FTA and the TLR4/NF-κB pathway was stronger than that of other components. All the results suggested that FTA, as a key component of FS, played an important role in alleviating cholestatic liver injury.

TLRs, a family of transmembrane receptors, play a crucial role in the innate immune system mainly because they can initiate innate immune responses by recognizing cell walls or specific nucleic acids of microbial pathogens. Once the molecular patterns related to pathogens and endogenous damage are recognized by TLRs, the signals are transmitted to the adapter molecule MyD88 to trigger the classic inflammatory cascade reaction, leading to excessive NF-κB activation [46]. This in turn leads to a series of cascade reactions inducing inflammation (including cytokines, chemokines, and adhesion molecules) and transcription of genes related to antimicrobial defense [47]. The liver is a crucial organ involved in bile synthesis and metabolism. When cholestasis occurs, a large amount of toxic bile acids will accumulate in the liver because the bile cannot be excreted from the liver. Eventually, these toxic bile acids lead to hepatic inflammation, which may further damage the liver. The entries for positive regulation of cell migration, tumor necrosis factor production, inflammatory response, cellular response to lipopolysaccharide, and interferon alpha production analyzed by GO bioprocess enrichment suggested that FTA may be more specific for inflammatory conditions.

Molecular docking outcomes revealed that FTA affected the structural foundation of TLR4 and NF-κB. While the Asn526 and Asn575 residues of TLR4 were mutated, TLR4 would eliminate the response to LPS and prevent cell surface expression [48]. FTA formed hydrogen bonds with the two residues and combined nearby residues, which may have similar effects. Variation in the Val61 residue of NF-κB can affect its binding activity to DNA [49,50]. The hydrophobic force between FTA and Val61 and other forces with nearby residues indicated that FTA may combine with the structural domain where NF-κB binds to DNA, and then competitively inhibit the binding activity of NF-κB to DNA.

Next, we established a mouse model of cholestasis induced by DDC and conducted in vivo validation experiments. The in vivo experimental results verified that FTA could not only effectively reduce liver inflammation in mice (mainly manifested by decreased mRNA expression of the liver inflammatory cytokines TNF-α, IL-1β, IL-6, and F4/80), but also alleviate systemic inflammation (mainly manifested by decreased levels of the serum inflammatory cytokines TNF--α, IL-1-β, and IL-6). More importantly, FTA intervention could also block the conduction of the TLR4/MYD88/NF-κB pathway, thus effectively preventing the liver from suffering from inflammatory damage.

In addition, cholestasis often accompanies the development of liver fibrosis, mainly due to impaired or interrupted bile production resulting in intracellular retention of toxic bile components, an excess of which will cause liver damage and fibrosis [51,52,53]. Liver fibrosis is usually accompanied by hepatic stellate cell (HSC) activation and promotes a phenotypic shift from a quiescent phenotype to an activated phenotype. The activated HSCs participate in the formation of liver fibrosis and the reconstruction of intrahepatic structures through the proliferation and secretion of extracellular matrix (ECM), while α-SMA is the sign of HSC activation [54,55]. Furthermore, there is evidence that MMP-2 plays a crucial role in modulating the synthesis and degradation of ECM [56,57]. The results of this study proved that FTA treatment could improve the liver pathology, function, and collagen deposition of mice. At the same time, WB and immunohistochemistry experiments confirmed that FTA could repress α-SMA protein expression, thus repressing HSC activation. In addition, FTA can also repress the MMP-2 gene and protein expression, thereby reducing the accumulation of ECM components. In a word, these results jointly confirmed that FTA significantly ameliorated liver fibrosis caused by cholestasis. 

Even though the TLR4/NF-κB pathway may be more important, network pharmacology results suggested that the role of other signaling pathways in the prevention and treatment of CLD by FS was also worth investigating. For example, as reported, the modulation of the PI3K-Akt signaling pathway has been shown to have a positive effect on the treatment of cholestasis [58,59,60,61,62]. Our previous studies have demonstrated that FTA may exert hepatoprotective effects by regulating the apoptosis pathway mediated by the PI3K-Akt signaling pathway [63]. Chronic liver injury caused by cholestasis may lead to anoxic areas in the liver that may induce HIF-1α activation, which further regulates a variety of fibrotic mediators, and stimulates the overproduction of collagen and liver fibrosis [64,65,66]. In the cholestasis of pregnancy, prolactin and its receptors are involved in water-salt metabolism and in turn, affect liver bile excretion [67,68,69]. Relaxin has natural anti-fibrosis activity in many organs, which can weaken the fibrosis characteristics of activated HSCs and reverse the formation of liver fibrosis [70,71,72]. Thereupon, the effects of FS on HIF-1, prolactin, and relaxin, and the relationships between these effects and cholestasis need to be validated and explored in more depth in the future.

## 5. Conclusions

**Figure 10:**
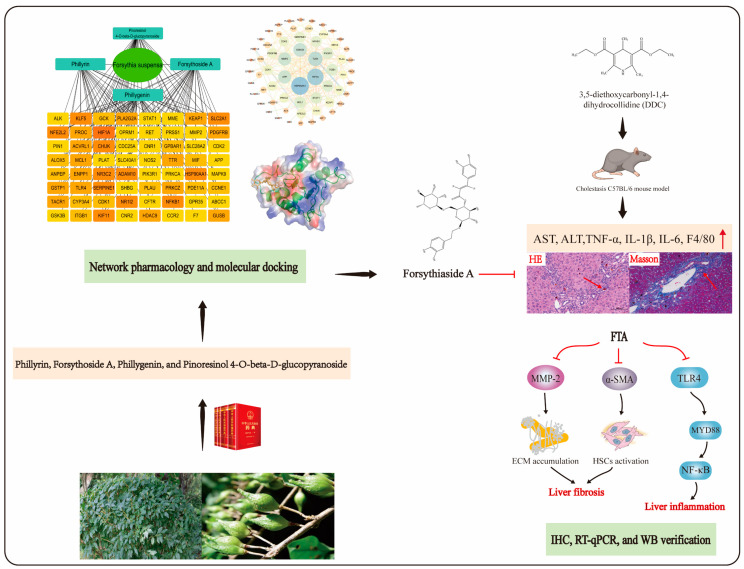
Experimental flow and molecular mechanism diagram in this study.

We revealed the potential molecular mechanisms of FS and its key components to prevent and treat cholestasis through network pharmacological methods and molecular docking techniques. Meanwhile, in vivo experiments showed that the key ingredient FTA could play an anti-inflammatory, hepatoprotective, and anti-fibrosis role by modulating the TLR4/NF-κB pathway, repressing HSC activation, ECM accumulation, and inflammatory factor release (Figure 10). All in all, these results could provide a scientific reference for the development of natural plant resources as drugs or functional foods to prevent and treat cholestasis.

## Figures and Tables

**Table 1:** The manufacturer and ID of reagents and chemicals.

Materials	Manufacturer	ID
FTA (purity above 98%)	Chroma Biotechnology Co., Ltd., Chengdu, China	79916-77-1
3,5-diethoxycarbonyl-1,4-dihydrocollidine (DDC)	Xiya Chemical Technology Co., Ltd., Shandong, China	632-93-9
Aspartate aminotransferase (AST) assay kit	Nanjing Jiancheng Bioengineering Institute, Jiangsu, China	C010-2-1
Alanine aminotransferase (ALT) assay kit		C009-2-1
Tumor necrosis factor-α (TNF-α) assay kit	Elabscience Biotechnology Co., Ltd., Wuhan, China	E-EL-M0037c
Interleukin (IL)-6 assay kit		E-EL-M0044c
IL-1β assay kit		E-EL-M3063
α-smooth muscle actin (α-SMA) antibody	Affinity, Cincinnati, OH, USA	AF1032
Matrix metalloproteinase 2 (MMP-2) antibody	ABclonal, Wuhan, China	A19080
F4/80 antibody		A18637
Toll-like receptor 4 (TLR4) antibody		A21626
Myeloid differentiation primary response 88 (MYD88) antibody		A21905
Nuclear factor kappa B (NF-κB) p65 antibody		A18210
*p*-NF-κB p65 antibody		AP0944
β-actin antibody	Servicebio, Wuhan, China	GB15001
Goat Anti-Rabbit IgG (H + L) HRP secondary antibody	Multi science, Hangzhou, China	GAR0072
Total RNA isolation kit	Foregene, Chengdu, China	RE-03014
HiScript^®^ IIQ RT SuperMix for qPCR	Vazyme, Nanjing, China	R223-01
ChamQ SYBR qPCR Master Mix		Q311-02

**Table 2:** Specific primers sequences used in RT-qPCR.

Gene	ID	Forward Primer (5′-3′)	Reverse Primer (5′-3′)
MMP-2	17390	CCATGTGTCTTCCCCTTCA	CCCCACTTCCGGTCATC
TNF-α	21926	GACAGTGACCTGGACTGTGG	TGAGACAGAGGCAACCTGAC
IL-1β	16176	GAAGAAGAGCCCATCCTCTG	TCATCTCGGAGCCTGTAGTG
IL-6	16193	CTGCAAGAGACTTCCATCCAG	AGTGGTATAGACAGGTCTGTTGG
F4/80	13733	TGGGAGCTACTTCTGCACT	AGGAGCCTGGTACATTGGT

**Table 3:** Basic information of four active ingredients of FS.

Component Name	PubChem CID	Molecular Formula	Canonical SMILES
Phillyrin	101712	C_27_H_34_O_11_	COC1=C(C=C(C=C1)C2C3COC(C3CO2)C4=CC(=C(C=C4)OC5C(C(C(C(O5)CO)O)O)O)OC)OC
Forsythoside A	5281773	C_29_H_36_O_15_	CC1C(C(C(C(O1)OCC2C(C(C(C(O2)OCCC3=CC(=C(C=C3)O)O)O)O)OC(=O)C=CC4=CC(=C(C=C4)O)O)O)O)O
Phillygenin	3083590	C_21_H_24_O_6_	COC1=C(C=C(C=C1)C2C3COC(C3CO2)C4=CC(=C(C=C4)O)OC)OC
Pinoresinol 4-O-beta-D-glucopyranoside	486614	C_26_H_32_O_11_	COC1=C(C=CC(=C1)C2C3COC(C3CO2)C4=CC(=C(C=C4)OC5C(C(C(C(O5)CO)O)O)O)OC)O

**Table 4:** Molecular docking results of four active components of FS in the treatment of cholestasis.

Gene Symbol	Protein	PDB ID	Resolution	Minimum Binding Free Energy (kcal/mol)			
				Forsythoside A	Phillygenin	Phillyrin	Pinoresinol 4-O-Beta-D-Glucopyranoside
TLR4	Toll-like receptor 4	4G8A	2.40 Å	−7.3	−6.5	−6.9	−6.8
NFKB1	Nuclear factor NF-kappa-B p105 subunit	1SVC	2.60 Å	−7.3	−6.5	−7.1	−7.3

**Table 5:** Molecular docking results of forsythoside A with MMP2, TLR4, MYD88, and IKBKB.

Gene Symbol	Protein	PDB ID	Resolution	Minimum Binding Free Energy (kcal/mol)
MMP2	72 kDa type IV collagenase	1CK7	2.80 Å	−8.7
TLR4	Toll-like receptor 4	4G8A	2.40 Å	−7.3
MYD88	Myeloid differentiation primary response protein MyD88	7BER	2.30 Å	−7.4
NFKB1	Nuclear factor NF-kappa-B p105 subunit	1SVC	2.60 Å	−7.3

## Data Availability

The data generated and/or analyzed in this study may be obtained from the corresponding author upon reasonable request.
